# The Alternative Oxidase AOX Does Not Rescue the Phenotype of *tko^25t^* Mutant Flies

**DOI:** 10.1534/g3.114.013946

**Published:** 2014-08-21

**Authors:** Kia K. Kemppainen, Esko Kemppainen, Howard T. Jacobs

**Affiliations:** * BioMediTech and Tampere University Hospital, FI-33014 University of Tampere, Tampere, Finland; † Research Program of Molecular Neurology, FI-00014 University of Helsinki, Helsinki, Finland

**Keywords:** mitochondrial disease, oxidative phosphorylation, gene therapy, seizures, developmental delay

## Abstract

A point mutation [*technical knockout^25t^* (*tko^25t^*)] in the *Drosophila* gene coding for mitoribosomal protein S12 generates a phenotype of developmental delay and bang sensitivity. *tko^25t^* has been intensively studied as an animal model for human mitochondrial diseases associated with deficiency of mitochondrial protein synthesis and consequent multiple respiratory chain defects. Transgenic expression in *Drosophila* of the alternative oxidase (AOX) derived from *Ciona intestinalis* has previously been shown to mitigate the toxicity of respiratory chain inhibitors and to rescue mutant and knockdown phenotypes associated with cytochrome oxidase deficiency. We therefore tested whether AOX expression could compensate the mutant phenotype of *tko^25t^* using the GeneSwitch system to activate expression at different times in development. The developmental delay of *tko^25t^* was not mitigated by expression of AOX throughout development. AOX expression for 1 d after eclosion, or continuously throughout development, had no effect on the bang sensitivity of *tko^25t^* adults, and continued expression in adults older than 30 d also produced no amelioration of the phenotype. In contrast, transgenic expression of the yeast alternative NADH dehydrogenase Ndi1 was synthetically semi-lethal with *tko^25t^* and was lethal when combined with both AOX and *tko^25t^*. We conclude that AOX does not rescue *tko^25t^* and that the mutant phenotype is not solely due to limitations on electron flow in the respiratory chain, but rather to a more complex metabolic defect. The future therapeutic use of AOX in disorders of mitochondrial translation may thus be of limited value.

*Drosophila* provides a useful animal model for human genetic diseases (Lloyd and Taylor 2010; Lu and Vogel 2009), including those associated with mitochondrial dysfunction (Sánchez-Martinez *et al.* 2006, Palladino 2010). Prominent among the latter are the many diseases caused by deficiency or malfunction of components of the machinery of mitochondrial protein synthesis (Pearce *et al.* 2013). These can be caused by point mutations of mitochondrial DNA (mtDNA), by large mtDNA deletions, or by nuclear gene lesions, and can involve interactions with environmental factors, including some antibiotics. Although their clinical phenotypes vary, a common thread is deficiency of multiple respiratory chain complexes, including ATP synthase, which include mtDNA-encoded subunits. The resulting metabolic crisis then produces a developmental and physiological disease condition, which can be widespread, severe, and often fatal.

We have previously investigated a *Drosophila* model of such diseases; *tko^25t^* carries a (recessive) point mutation in the gene for mitoribosomal protein S12 (Royden *et al.* 1987; Shah *et al.* 1997). *tko^25t^* flies exhibit developmental delay, sensitivity to seizures induced by mechanical stress (“bang sensitivity”), and a set of linked phenotypes that share features with human mitochondrial disease, including hearing impairment and sensitivity to antibiotics that impair mitochondrial protein synthesis (Toivonen *et al.* 2001). At the molecular level, *tko^25t^* shows decreased abundance of mitoribosomal small subunits, multiple respiratory chain and ATP synthase deficiency (Toivonen *et al.* 2001), and altered gene expression indicative of a metabolic shift toward glycolytic lactate production and anaplerotic pathways (Fernández-Ayala *et al.* 2010).

The phenotype of *tko^25t^* flies can be partially suppressed by segmental duplication of the mutant gene in its natural chromosomal milieu (Kemppainen *et al.* 2009), by cybridization to specific suppressor cytoplasmic (mtDNA) backgrounds (Chen *et al.* 2012), or by overexpression of *spargel* (Chen *et al.* 2012), the *Drosophila* homolog of PGC1-α, proposed to function as a master regulator of mitochondrial biogenesis (Scarpulla 2011). In other studies, we found that toxic inhibition of complex III (cIII) by antimycin or cIV by cyanide, or phenotypes resulting from mutations or knockdown of cIV subunits or the cIV assembly factor *Surf1* in *Drosophila*, could be mitigated by concomitant expression of the mitochondrial alternative oxidase (AOX) from **Ciona* intestinalis* (Fernández-Ayala *et al.* 2009; Kemppainen *et al.* 2014).

AOX is widespread in eukaryotes, being found in plants, fungi, and many animal phyla, although not in arthropods or vertebrates (McDonald *et al.* 2009). It provides a nonproton-translocating bypass of the cytochrome segment of the mitochondrial respiratory chain, maintaining electron flow under conditions in which it would be inhibited by high membrane potential, toxic inhibition, or insufficient capacity of cIII and/or cIV. *tko^25t^* flies exhibit multiple respiratory chain deficiency, including profoundly decreased activity of both cIII and cIV (Toivonen *et al.* 2001). However, whereas lactate dehydrogenase can theoretically compensate, at least in part, for the lack of cI (Fernández-Ayala *et al.* 2010), ubiquinone-linked dehydrogenases, such as succinate dehydrogenase (complex II, cII), require the cytochrome chain for onward electron transfer to oxygen to reoxidize ubiquinol. Thus, even though it cannot directly support ATP production, AOX expression in *tko^25t^* should facilitate intermediary metabolism, leading to an amelioration of the mutant phenotype if that phenotype is due to limitations on electron flow through cIII and cIV.

We therefore set out to test whether expression of *Ciona* AOX in *Drosophila* at different times in the life-cycle could correct the major organismal phenotypes of *tko^25t^*, namely bang sensitivity and developmental delay.

## Materials and Methods

### Flies, maintenance, and behavioral assays

*Drosophila* lines were as described previously (Toivonen *et al.* 2001; Fernández-Ayala *et al.* 2009; Sanz *et al.* 2010a). Flies were maintained at 25° on standard medium with supplements, as previously described (Fernández-Ayala *et al.* 2009), including RU486 (Mifepristone), with indicated time to eclosion and bang sensitivity at 25° measured as previously described (Toivonen *et al.* 2001).

### RNA isolation and analysis

RNA extraction and QRTPCR were performed as previously described (Fernández-Ayala *et al.* 2009). RNA isolations were performed in triplicate from batches of 40 males or 30 virgin females. For QRTPCR, cDNA was synthesized using High-Capacity cDNA Reverse-Transcription kit (Life Technologies, Carlsbad, CA). Analysis used a StepOnePlus instrument (Life Technologies) with the manufacturer’s SYBR Green PCR reagents and customized AOX primers and normalization to *RpL32* RNA as previously described (Fernández-Ayala *et al.* 2009).

### Metabolic assays

ATP levels in adult female flies were measured as previously described (Chen *et al.*, 2012), along with ATP standards. Mitochondrial reactive oxygen species (ROS) production was measured essentially according to Ballard *et al.* (2007) as hydrogen peroxide produced in whole-body mitochondrial extracts from 2- to 5-d-old females using a substrate mix of 5 mM pyruvate, 5 mM proline, 20 mM sn-glycerol-3-phosphate, and 1 mM ADP.

## Results

### Transgenic expression of AOX in *Drosophila* using an inducible driver

**Figure 1:**
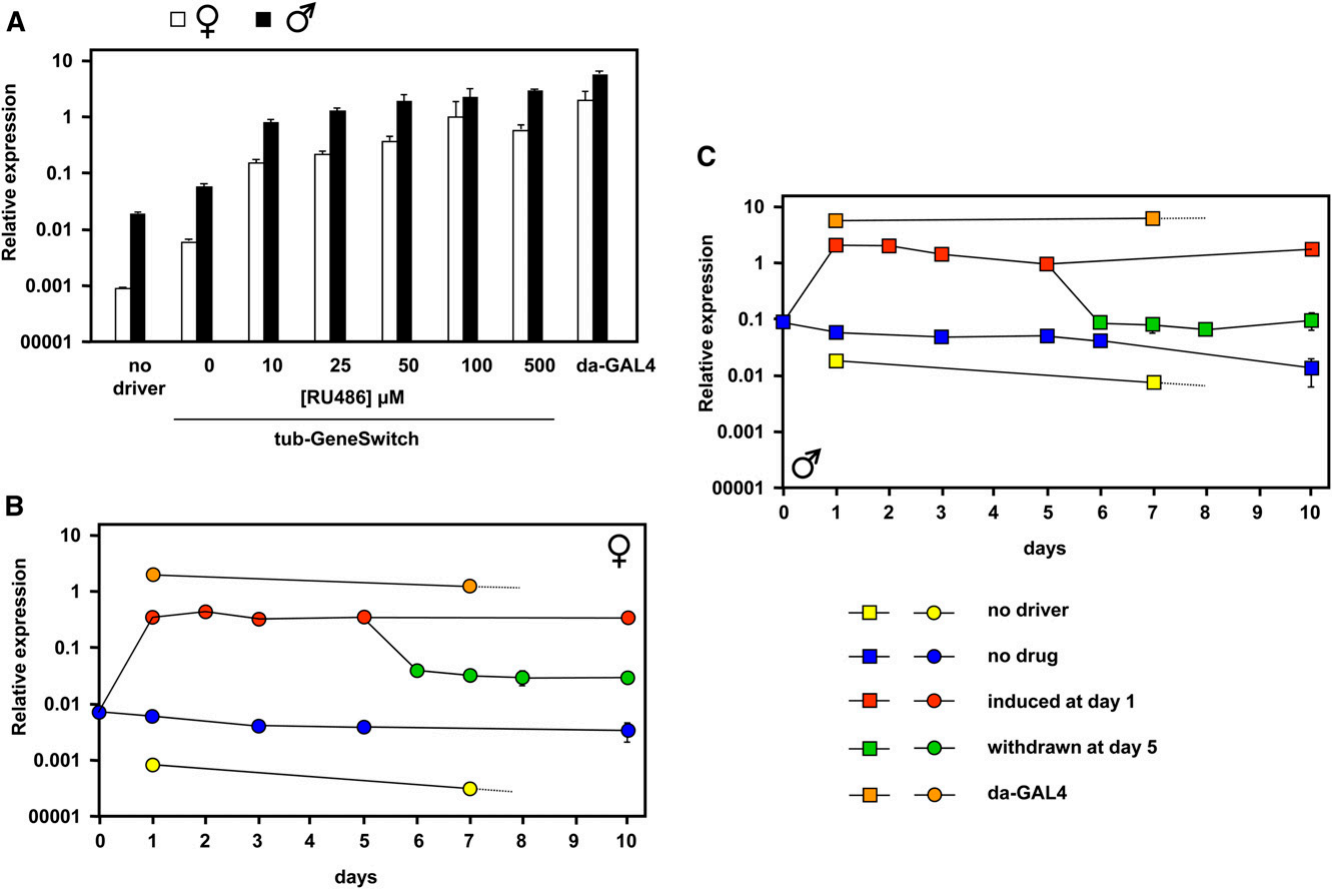
AOX expression in adult flies driven by tubulin-GeneSwitch. (A) Relative AOX expression, determined by QRTPCR normalized to *RpL32* control RNA, in 2-d-old UAS-AOX flies bearing the drivers indicated exposed to different concentrations of RU486 for 24 hr. Means ± SD of three biological replicates. Note the logarithmic scale. (B and C) Relative AOX expression in adult UAS-AOX flies bearing the indicated drivers and exposed to 200 μM RU486 as shown. Means ± SD of three biological replicates.

We previously documented the amount of expression of AOX at the RNA level in transgenic flies containing single and double copies of the UAS-AOX transgene activated by different ubiquitously acting drivers (Fernandez-Ayala *et al.* 2009). In the same study, using the drug-inducible tubulin-GeneSwitch driver (tub-GS), we determined the minimal level of the inducing drug RU486 (10 μM) that would sustain maximal AOX expression throughout development when flies were cultured in drug-containing food. To be able to induce and sustain AOX expression at different times during adult life, we first conducted further tests using the tub-GS driver (Figure 1). Expression of AOX was induced in 1-d-old adults using different concentrations of RU486 and was measured 24 hr later using UAS-AOX–bearing flies with no driver or with the highly active da-GAL4 driver as controls (Figure 1A). Even without drug, the tub-GS driver supported AOX expression at a three-fold to 10-fold higher level than in the absence of any driver. As observed previously using various drivers (Fernandez-Ayala *et al.* 2009), expression in males was always approximately three-fold higher than in females, which is probably a feature of the standard UAS transgenic construct and/or dosage compensation elements associated with the linked mini-white marker gene. RU486 even at low doses increased expression at least 10-fold further, and expression reached a plateau at a drug concentration of 100 μM. To be sure of fully activating expression, we thereafter routinely used 200 μM RU486 as the activating condition.

Next, we determined the kinetics of induced expression and the effects of sustained drug exposure or its withdrawal (Figure 1, B and C). AOX expression already reached a plateau level after 1 d of drug exposure in females (Figure 1B) and males (Figure 1C); thereafter, it remained constant if flies were maintained on drug-containing food. If drug was withdrawn by switching to drug-free food at day five, then expression decreased to a new plateau level by 1 d later. However, this level was two-fold to three-fold higher than that of flies never exposed to drug. Flies endowed with UAS-AOX and tub-GS were cultured continuously on RU486-containing food for many weeks and remained phenotypically indistinguishable from flies grown on drug-free food.

### Adult-specific induction of AOX does not rescue bang sensitivity of *tko^25t^*

**Figure 2:**
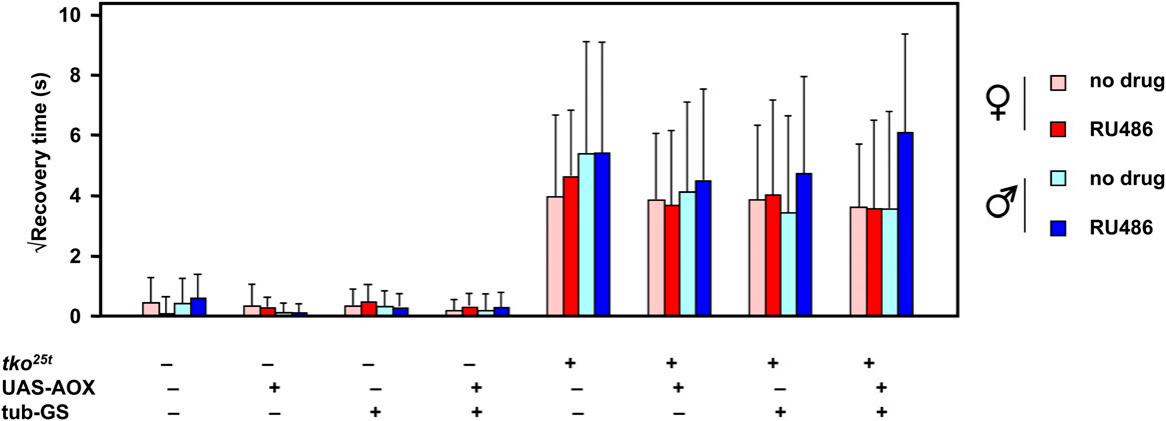
Bang sensitivity is unaffected by AOX induction in adult flies. Bang sensitivity (square-root of recovery time from vortexing) of 2-d-old flies of the sex and genotype indicated, with or without 24 hr of prior treatment with 200 μM RU486. Means ± SD for groups of 30 individually analyzed flies.

Bang sensitivity is generally considered to arise from a functional defect of nerve conduction during high-frequency stimulation in the giant fiber pathway (Pavlidis and Tanouye 1995; Lee and Wu 2002; Fergestad *et al.* 2006; Ueda *et al.* 2008). Bang-sensitive mutants with an underlying mitochondrial defect, including *kdn* (citrate synthase) and *sesB^1^* (adenine nucleotide translocase) as well as *tko^25t^* display a characteristic seizure pattern (Fergestad *et al.* 2006). We therefore decided to test whether expression of AOX in *tko^25t^* mutant flies could compensate for the mitochondrial defect and thus alleviate bang sensitivity. We crossed tub-GS into the *tko^25t^* background using a balancer chromosome strategy to analyze progeny from a single experimental cross that generated flies carrying *tko^25t^*, tub-GS, and/or UAS-AOX in all eight possible combinations. Bang sensitivity was tested in 2-d-old males and females of each class, either with or without transfer 24 hr earlier to food containing 200 μM RU486 (Figure 2). Unambiguously, the results indicate that AOX is unable to modify the bang-sensitive phenotype of *tko^25t^* adults, and it does not induce any detectable bang sensitivity in control flies. In fact, applying Student’s *t* test with Bonferroni correction confirmed that there were no significant differences between any of the classes that were mutant for *tko^25t^*, irrespective of sex, transgene, driver, or RU486 induction. Similarly, there were no significant differences between any of the classes that were wild-type for the *tko* gene, irrespective of these other parameters. As expected, the difference between *tko^25t^* mutant flies of each class and the corresponding class without *tko^25t^* was significant (*P* < 0.01) in every case.

### Continuous induction of AOX throughout development does not rescue *tko^25t^*

Considering an alternative hypothesis, that the bang-sensitive phenotype of *tko^25t^* is established during development, we conducted similar crosses but used fly food containing RU486. In our previous study (Fernandez-Ayala *et al.* 2009), we established that 10 μM RU486 was sufficient to induce maximal transgene expression during the larval stages, so we used this concentration of the drug along with drug-free control vials. This procedure allowed us also to analyze effects on the second canonical phenotype of *tko^25t^*, developmental delay, which was previously found to occur uniquely during the larval (growth) stages (Toivonen *et al.* 2001).

**Figure 3:**
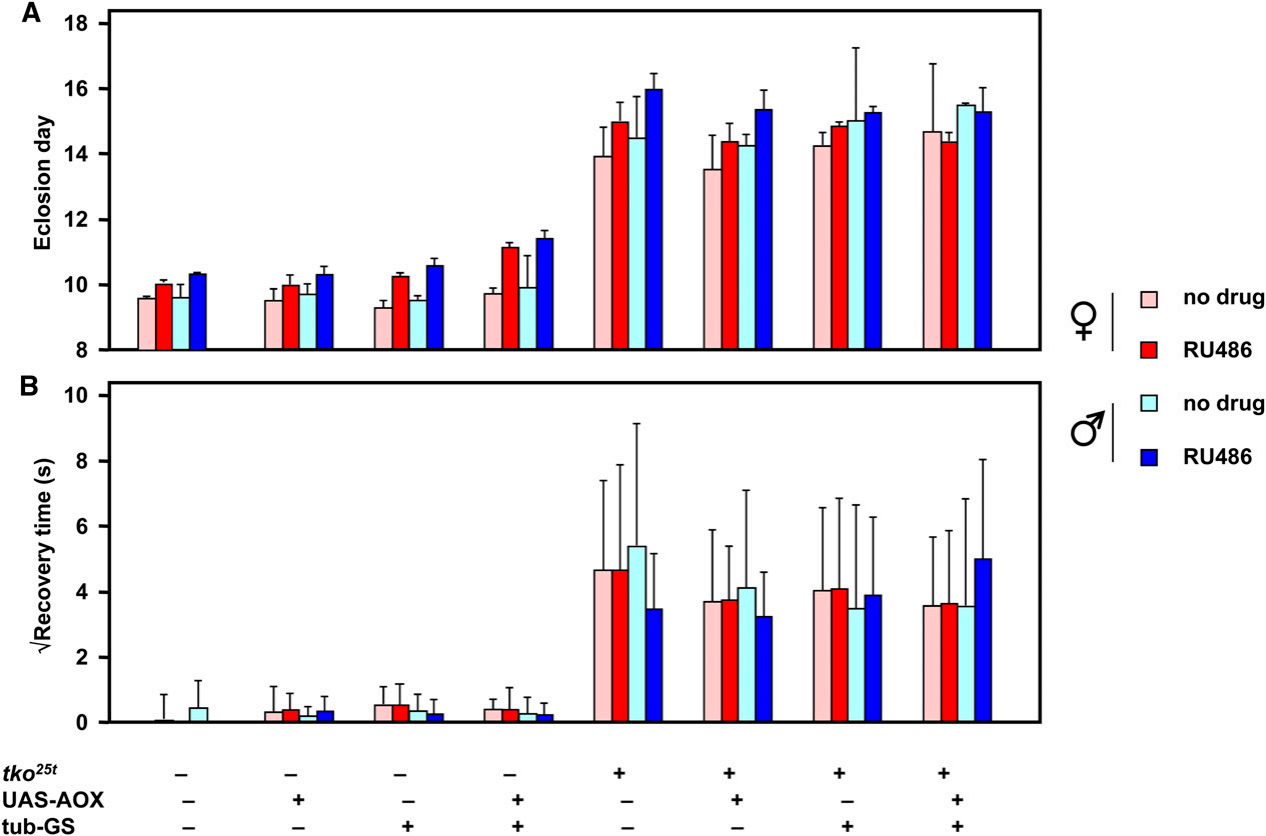
Phenotype of *tko^25t^* is unaffected by AOX expression throughout development. (A) Eclosion day and (B) bang sensitivity of 1-d-old flies of the sex and genotype indicated, cultured throughout development on medium with or without 10 μM RU486. Means ± SD based on eclosion data from three replicate experiments and bang sensitivity of groups of 50 individually analyzed flies.

Once again, we observed no rescue of the mutant phenotype that was attributable to AOX expression (Figure 3). The developmental delay of *tko^25t^* mutant flies (Figure 3A) was slightly greater in males than in females, as observed previously (Kemppainen *et al.* 2009), and an additional delay of approximately 1 d was produced in flies of all genotypes and both sexes by the presence of RU486 in the food. The UAS-AOX transgene, the tub-GS driver, and the two in combination did not produce any significant change in developmental timing of *tko^25t^* mutant flies, although there was a slight delay produced by AOX expression in wild-type flies, as reported previously using the da-GAL4 driver. The bang sensitivity of the progeny flies showed no significant change according to any of the parameters tested, except for the presence of the *tko^25t^* mutation itself (Figure 3B).

### Prolonged adult induction of AOX does not rescue bang sensitivity of *tko^25t^*

To test whether correction of the *tko^25t^* phenotype in adult flies requires long-term expression of AOX, we cultured *tko^25t^* flies generated in the previous crosses continuously for a period of 30 d on food either with or without RU486 at the inducing concentration of 200 μM, noting the previous result that sustained expression requires continuous exposure to the drug. This also enabled us to check the stability of the phenotype during adult life, which, to our knowledge, has not previously been studied systematically.

**Figure 4:**
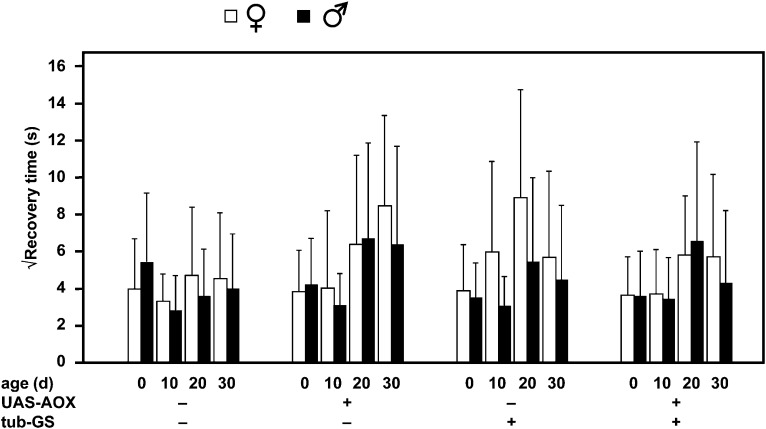
Bang sensitivity is unaffected by continuous AOX expression over 30 d. Bang sensitivity of flies of the sex, genotype, and age indicated, with or without continuous growth as adults on media containing 200 μM RU486. Means ± SD for groups of 50 individually analyzed flies.

Bang sensitivity was unaffected by any of the parameters tested in this experiment (Figure 4). There was no rescue (or worsening) of the phenotype either by basal or by induced AOX expression, no effect of age, no difference between the sexes, and no effect of tub-GS.

### Ndi1 expression during development is lethal to *tko^25t^*

Because AOX expression at any stage of the fly life-cycle had no effect on the major phenotypic features of *tko^25t^* mutants, we considered the hypothesis that the steps in mitochondrial electron flow that AOX bypasses may not be crucial determinants of the phenotype. The *tko^25t^* mutation impacts all four of the enzymatic complexes of the oxidative phosphorylation (OXPHOS) system that contain mitochondrial translation components (Toivonen *et al.* 2001), but it is unclear which is limiting for respiration or ATP synthesis. Because complex I (cI) activity is severely affected by the mutation, we considered the alternative hypothesis that a decreased capacity for electron flow through cI alone underlies the *tko^25t^* mutant phenotype, and that decreased capacity of complexes III and/or IV is immaterial, thus accounting for a failure of AOX expression to modify the phenotype.

**Figure 5:**
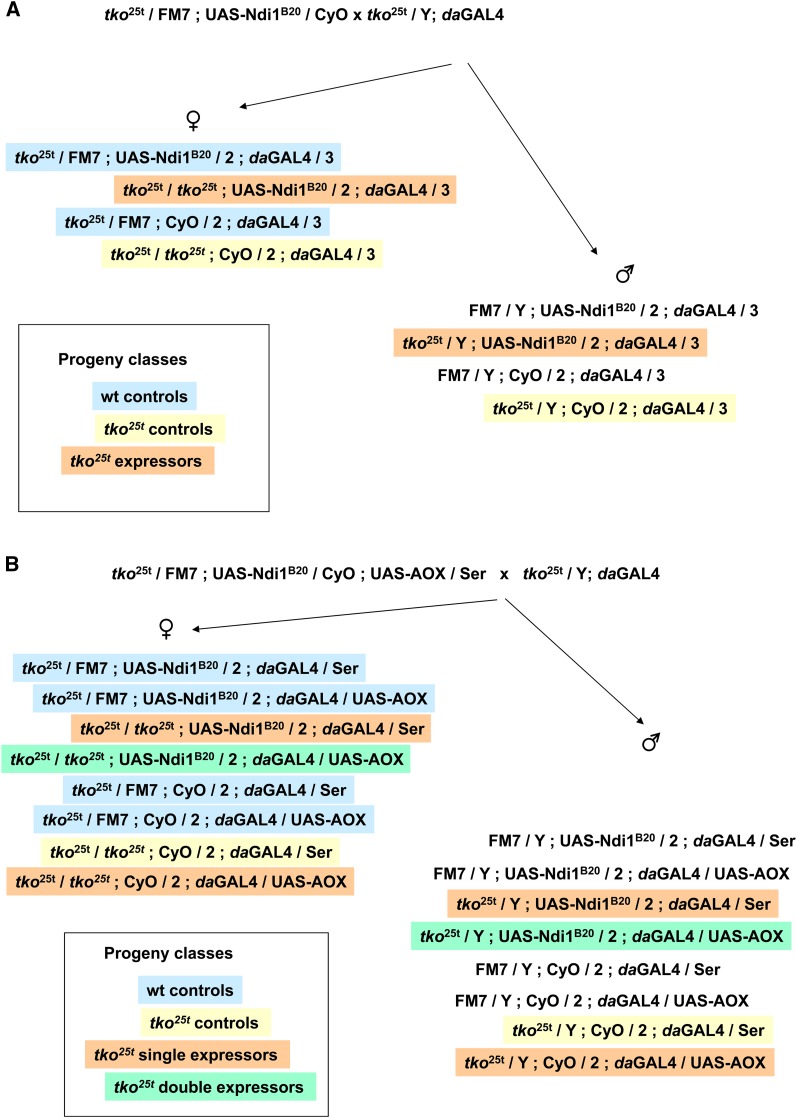
Genetic crosses used to test rescue of *tko^25t^*. Crosses used to test rescue by (A) Ndi1 or (B) Ndi1 plus AOX combined. Progeny classes are color-coded as indicated to denote their meaning in the experiment. The results of the cross are shown in Table 1. Note that FM7 / Y males do not contain an unmanipulated X-chromosome, so they are not strictly a wild-type control.

To test this idea, we set-up a genetic cross (Figure 5A) to investigate whether an analogous bypass of cI using the nonproton-pumping NADH dehydrogenase from yeast (Ndi1) could rescue the phenotype. Ndi1 expression was shown previously to be benign in *Drosophila* and to rescue the lethality of severe knockdown of cI subunits (Sanz *et al.* 2010b). We introduced the ubiquitously acting da-GAL4 driver and a UAS-Ndi1 transgene separately into the *tko^25t^* mutant strain and then crossed females heterozygous both for *tko^25t^* and UAS-Ndi1 with *tko^25t^* males carrying da-GAL4 (Figure 5A). The cross repeatedly gave a low number of *tko^25t^* progeny (Table 1). However, almost all of them carried the balancer marker in place of UAS-Ndi1, indicating that the combination of da-GAL4, *tko^25t^* and UAS-Ndi1 is semi-lethal. Expression of Ndi1 in *tko^25t^* heterozygotes had a far less dramatic effect. We conclude that, far from rescuing *tko^25t^*, expression of Ndi1 is selectively deleterious to *tko^25t^* mutant flies.

**Table 1:** Test of ability of Ndi1 expression to rescue *tko*^25t^

**Genotype***^a^*	**Sex**	**Number of Progeny***^b^*
*tko*^25t^ / FM7 ; CyO / 2 ; *da*GAL4 / 3	Female	152
*tko*^25t^ / FM7 ; UAS-Ndi1^B20^ / 2 ; *da*GAL4 / 3	Female	72
*tko*^25t^ / *tko^25t^* ; CyO / 2 ; *da*GAL4 / 3	Female	57
*tko*^25t^ / *tko^25^*^t^ ; UAS-Ndi1^B20^ / 2 ; *da*GAL4 / 3	Female	1
FM7 / Y ; CyO / 2 ; *da*GAL4 / 3	Male	65
FM7 / Y ; UAS-Ndi1^B20^ / 2 ; *da*GAL4 / 3	Male	25
*tko*^25t^ / Y ; CyO / 2 ; *da*GAL4 / 3	Male	20
*tko*^25t^ / Y ; UAS-Ndi1^B20^ / 2 ; *da*GAL4 / 3	Male	1

a Output from cross shown in Fig. 5A.

b A repeat experiment gave similar results.

This result raises the possibility that although neither Ndi1 nor AOX can individually rescue *tko^25t^*, the co-expression of both transgenes might do so. This would be the case, for example, if the *tko^25t^* phenotype were due to a combined limitation on electron flow at both cI and at cIII+cIV of similar magnitude. Although co-expression of Ndi1 and AOX at 25° was previously shown to be synthetically lethal even in wild-type flies (Sanz *et al.* 2010b), in trial experiments we were able to obtain co-expressing flies when cultured at 18°. We therefore implemented the experimental cross illustrated in Figure 5B to determine whether Ndi1 and AOX co-expression can rescue *tko^25t^*. As shown in Table 2, although control flies were now obtained, and again there were only a few Ndi1-expressing flies in the *tko^25t^* mutant background, no doubly expressing *tko^25t^* flies eclosed. We conclude that, far from rescuing *tko^25t^*, combined expression of the two transgenes is more deleterious than of either alone.

**Table 2:** Test of ability of Ndi1 and AOX co-expression to rescue *tko*^25t^

**Genotype***^a^*	**Sex**	**Number of Progeny***^b^*
*tko*^25t^ / FM7 ; CyO / 2 ; *da*GAL4 / Ser	Female	54
*tko*^25t^ / FM7 ; CyO / 2 ; *da*GAL4 / UAS-AOX	Female	48
*tko*^25t^ / FM7 ; UAS-Ndi1^B20^ / 2 ; *da*GAL4 / Ser	Female	34
*tko*^25t^ / FM7 ; UAS-Ndi1^B20^ / 2 ; *da*GAL4 / UAS-AOX	Female	35
*tko*^25t^ / *tko^25t^* ; CyO / 2 ; *da*GAL4 / Ser	Female	23
*tko*^25t^ / *tko^25t^* ; CyO / 2 ; *da*GAL4 / UAS-AOX	Female	17
*tko*^25t^ / *tko^25^*^t^ ; UAS-Ndi1^B20^ / 2 ; *da*GAL4 / Ser	Female	5
*tko*^25t^ / *tko^25^*^t^ ; UAS-Ndi1^B20^ / 2 ; *da*GAL4 / UAS-AOX	Female	0
FM7 / Y ; CyO / 2 ; *da*GAL4 / Ser	Male	26
FM7 / Y ; CyO / 2 ; *da*GAL4 / UAS-AOX	Male	27
FM7 / Y ; UAS-Ndi1^B20^ / 2 ; *da*GAL4 / Ser	Male	8
FM7 / Y ; UAS-Ndi1^B20^ / 2 ; *da*GAL4 / UAS-AOX	Male	7
*tko*^25t^ / Y ; CyO / 2 ; *da*GAL4 / Ser	Male	19
*tko*^25t^ / Y ; CyO / 2 ; *da*GAL4 / UAS-AOX	Male	14
*tko*^25t^ / Y ; UAS-Ndi1^B20^ / 2 ; *da*GAL4 / Ser	Male	6
*tko*^25t^ / Y ; UAS-Ndi1^B20^ / 2 ; *da*GAL4 / UAS-AOX	Male	0

a Output from cross shown in Fig. 5B.

b A repeat experiment gave similar results.

### Effects on ATP or ROS do not correlate with modulation of *tko^25t^* phenotype

In previous studies we found decreased steady-state ATP levels in extracts from *tko^25t^* mutant flies, as well as elevated production of ROS in isolated *tko^25t^* mitochondria (Chen *et al.* 2012). However, the relevance of these observations to the organismal phenotype remains to be conclusively demonstrated. The effects of AOX and Ndi1 expression on the *tko^25t^* phenotype provided an opportunity to test this relationship further. To obtain a sufficient number of *tko^25t^* flies expressing Ndi1 to conduct this experiment, flies were reared at 18° instead of 25° (see previous section).

**Figure 6:**
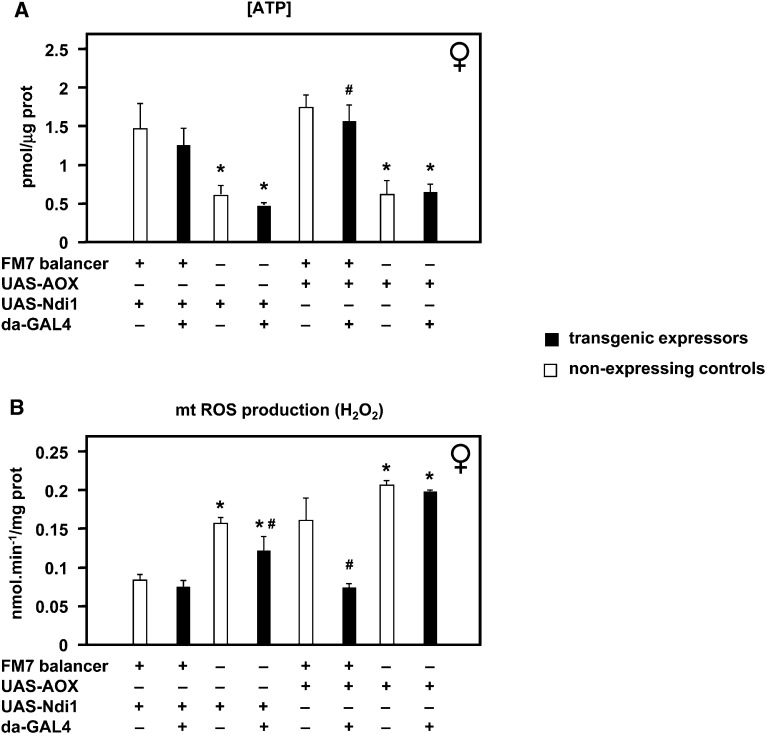
Altered ATP and ROS levels do not account for phenotypic effects of AOX or Ndi1. Effects of Ndi1 and AOX expression on (A) ATP levels and (B) mitochondrial ROS production of female *tko^25t^* flies of the indicated genotypes, reared at 18°. Flies were homozygous for *tko^25t^*, except those carrying the FM7 balancer, which are phenotypically wild-type. Means ± SD for three or more biological replicates of each genotype.*Significant differences between *tko^25t^* homozygotes and heterozygotes of otherwise identical genotypes, *P* < 0.01, Student’s *t* test, two-tailed. #Significant differences between Ndi1 or AOX expressors and nonexpressors of otherwise identical genotypes, *P* < 0.05, Student’s *t* test, two-tailed.

We confirmed the previous observation of decreased ATP levels in *tko^25t^* homozygotes compared with heterozygous controls (Figure 6A) but found no significant alteration thereof when either AOX or Ndi1 was expressed. Mitochondrial ROS production in *tko^25t^* homozygotes was also elevated in every case compared with heterozygous controls (Figure 6B). This was unaffected by expression of AOX but modestly alleviated by Ndi1 expression, despite the fact that the effect of Ndi1 on the overall organismal phenotype was deleterious. This, plus the wide variation in ROS production according to genetic background (reflecting different balancer chromosomes), implies that the *tko^25t^* organismal phenotype is also not directly determined by ROS.

## Discussion

In this work we set out to determine whether AOX from *Ciona intestinalis* can ameliorate the mutant phenotype of *tko^25t^*, which carries a mutation in mitoribosomal protein S12, resulting in globally decreased OXPHOS capacity. We found that induced AOX expression, whether during development, in freshly eclosed adults, or maintained in adults over a period of 30 d, has no effect on *tko^25t^*, nor does it produce a phenocopy of *tko^25t^* in wild-type flies. In contrast, ubiquitous expression of Ndi1, the alternative NADH dehydrogenase from yeast, was highly deleterious to *tko^25t^* during development and was lethal when combined with both *tko^25t^* and AOX.

### Failure of AOX rescue suggests that a complex metabolic defect underlies the *tko^25t^* phenotype

*tko^25t^* exhibits a functional deficiency of all four OXPHOS complexes containing mitochondrial translation products (Toivonen *et al.* 2001), but it is unclear which of these is limiting for electron transfer. Because AOX provides a functional bypass of complexes III and IV, its failure to rescue the organismal phenotype can be interpreted in one of several ways. The first would be that the residual activity of cIII/cIV is not limiting for mitochondrial electron transport in *tko^25t^*, and that the phenotype is entirely due to cI dysfunction. The second postulates that AOX is unable to rescue *tko^25t^* because, as a nonproton-motive enzyme, it does not support the synthesis of ATP, and ATP deficiency is what underlies the mutant phenotype. A third possibility is that the phenotype is a consequence of one or more processes on which AOX does not impinge, such as elevated ROS production, or proteotoxicity due to the protein synthesis defect. Although none of these can be entirely eliminated, the fact that Ndi1 expression worsens the phenotype, either alone or in combination with AOX, and that changes in ATP level or mitochondrial ROS production do not correlate with it, suggest that the mutant phenotype is determined either by a complex interplay of factors or by other metabolic effects that are as yet unknown. Disrupted redox homeostasis resulting from a cI defect should be rescuable by Ndi1. A combined limitation on electron flow at cI and cIII and/or cIV should be alleviated by combined expression of Ndi1 and AOX. Manifestly, these predictions are inconsistent with our findings.

Ndi1 is constitutively active (Sanz *et al.* 2010b), consistent with the fact that in its natural setting (in budding yeast) cI is absent. By diverting electrons away from cI, it may act to decrease net ATP production still further, but this seems unlikely to be the explanation for its effect on *tko^25t^* because the apparent additional decrease in ATP level (Figure 6A) was modest and not statistically significant. However, the low number of successfully eclosing flies may represent the tail of a distribution, with those individuals suffering further ATP depletion simply unable to complete development. Effects on mitochondrial ROS production also did not correlate with the organismal phenotype. Although we confirmed elevated ROS production in *tko^25t^* flies (Figure 6B), it was more affected by genetic background than by the expression of the alternative respiratory chain enzymes, and the effect of Ndi1 was again paradoxical. Note, however, that all metabolic assays were conducted on materials from flies reared at 18°, whereas for most of the phenotypic experiments reported here flies were cultured at 25°. This may have some bearing on the findings.

Proteotoxicity due to imbalance between cytosolic and mitochondrial protein synthesis has been implicated as a longevity mechanism, acting hormetically via the induction of the mitochondrial unfolded protein response (Houtkooper *et al.* 2013; Arnsburg and Kirstein-Miles 2014). However, decreased levels of NAD+ are associated with a failure of this mechanism (Mouchiroud *et al.* 2013). The deleterious effect produced by Ndi1 expression is again not consistent with this being the primary mechanism underlying the *tko^25t^* phenotype.

The failure of AOX to rescue bang sensitivity and developmental delay in *tko^25t^* reflects a similar finding for a second mutant affecting mitochondrial ATP production, *sesB^1^* (Vartiainen *et al.* 2014). *sesB^1^* carries a mutation in the gene encoding the major adult isoform of the adenine nucleotide translocase (Zhang *et al.* 1999) and, like *tko^25t^*, *sesB^1^* mutant flies show decreased steady-state ATP levels as well as bang sensitivity and developmental delay (Vartiainen *et al.* 2014). For these reasons, as well as the arguments stated above, we feel the “ATP hypothesis” cannot be entirely discounted, although other metabolic effects need to be further investigated as well.

### Bang sensitivity of *tko^25t^* is a developmental rather than a degenerative phenotype

Bang sensitivity is a commonly observed mutant phenotype in *Drosophila* and is due to lesions affecting a variety of cellular or physiological pathways, including, in addition to mitochondrial protein synthesis, adenine nucleotide transport and the TCA cycle (Fergestad *et al.* 2006), phospholipid metabolism (Pavlidis *et al.* 1994), ion pumps and channels (Schubiger *et al.* 1994; Kane *et al.* 2000; Iovchev *et al.* 2002; Parker *et al.* 2011), and proteolysis (Zhang *et al.* 2002). Although they manifest some similarities in their electrophysiological defects (Engel and Wu 1994), they fall into two classes depending on whether motor neurons are directly affected (Fergestad *et al.* 2006). Some of them show a clear degenerative phenotype with drastically shortened lifespan, whereas others, including *tko^25t^*, show only a modestly decreased lifespan and associated neuropathology (Fergestad *et al.* 2008). In the current study, we found no significant change in the bang sensitivity of *tko^25t^* over 30 d of adult life, in contrast to the synergistic and progressive effects on bang sensitivity seen when *tko^25t^* is combined with other bang-sensitive mutants (Fergestad *et al.* 2008). We conclude that the bang sensitivity of *tko^25t^* is a developmentally determined phenotype, at least in an otherwise wild-type genetic background

### Therapeutic implications for AOX in mitochondrial disease

AOX has been proposed as a therapeutic tool relevant to a wide variety of mitochondrial disorders (El-Khoury *et al.* 2014). The present work indicates important limitations of this concept, whatever the precise link between mitochondrial translational dysfunction and the organismal phenotype in *tko^25t^*. Despite profound effects on flies exposed to toxins or mutations directly or indirectly affecting cytochrome oxidase (Fernandez-Ayala *et al.* 2009; Kemppainen *et al.* 2014), or even the pleiotropic phenotypes caused by partial knockdown of DNA polymerase γ (Humphrey *et al.* 2012), AOX expression produced no detectable modification to the *tko^25t^* phenotype.

*tko^25t^* has been considered as a model for mitochondrial diseases, exhibiting not only seizures and developmental delay but also hearing impairment (Toivonen *et al.* 2001). It is of particular relevance to those disorders where the primary defect is in the mitochondrial translation system, which applies to many of the commonest pathological mtDNA mutations such as the 3243G > A MELAS mutation, as well as an increasingly recognized subset of nuclear gene mitochondrial disorders exhibiting multiple OXPHOS deficiencies (Pearce *et al.* 2013). The implementation of respiratory chain bypasses such as AOX or Ndi1 should, in theory, alleviate pathological phenotypes associated with restrictions on electron transport, depending on which segments of the respiratory chain are affected. In cases where multiple OXPHOS complexes are affected, both bypasses in combination might be needed to restore electron flow. *tko^25t^* constitutes a model for such diseases, yet neither AOX nor Ndi1 ameliorated the phenotype, and Ndi1 was even deleterious. As already indicated, Ndi1 and AOX do not restore proton pumping at the respiratory chain segments that they bypass, nor can they alleviate, *a priori*, all other aspects of mitochondrial dysfunction. Their uses in eventual therapy for disorders of mitochondrial translation therefore may be limited and clearly requires a fuller understanding of the pathophysiological mechanism case by case.
